# Use and Acceptability of HIV Self-Testing Among First-Time Testers at Risk for HIV in Senegal

**DOI:** 10.1007/s10461-019-02552-2

**Published:** 2019-06-13

**Authors:** Carrie E. Lyons, Karleen Coly, Anna L. Bowring, Benjamin Liestman, Daouda Diouf, Vincent J. Wong, Gnilane Turpin, Delivette Castor, Penda Dieng, Oluwasolape Olawore, Scott Geibel, Sosthenes Ketende, Cheikh Ndour, Safiatou Thiam, Coumba Touré-Kane, Stefan D. Baral

**Affiliations:** 1 Department of Epidemiology, Center for Public Health and Human Rights, Johns Hopkins School of Public Health, 615 N Wolfe St., Baltimore, MD 21205 USA; 2 Enda Sante, Dakar, Senegal; 3 Office of HIV/AIDS, Bureau for Global Health, USAID, Arlington, VA USA; 4 Division de La Lutte Contre Le Sida et Les IST, Ministry of Health, Dakar, Senegal; 5 Conseil National de Lutte contre le Sida, Dakar, Senegal; 6 Population Council, Washington, DC USA; 7 Laboratoire de Bacteriologie-Virologie, CHU Aristide Le Dantec, Dakar, Senegal

**Keywords:** HIV, Self-Testing, Key populations, Senegal, Sub-Saharan Africa

## Abstract

HIV Self-Testing (HIVST) aims to increase HIV testing coverage and can facilitate reaching the UNAIDS 90-90-90 targets. In Senegal, key populations bear a disproportionate burden of HIV and report limited uptake of HIV testing given pervasive stigma and criminalization. In these contexts, HIVST may represent a complementary approach to reach populations reporting barriers to engagement with existing and routine HIV testing services. In this study, 1839 HIVST kits were distributed in Senegal, with 1149 individuals participating in a pre-test questionnaire and 817 participating in a post-test questionnaire. Overall, 46.9% (536/1144) were first-time testers and 26.2% (300/1144) had tested within the last year; 94.3% (768/814) reported using the HIVST, and 2.9% (19/651) reported a reactive result which was associated with first-time testers (p = 0.024). HIVST represents an approach that reached first-time testers and those who had not tested recently. Implementation indicators suggest the importance of leveraging existing community structures and programs for distribution.

## Resumen

El autodiagnóstico del VIH (ADVIH) busca incrementar la cobertura de las pruebas del VIH y puede facilitar el alcance de los objetivos 90-90-90 de ONUSIDA. En Senegal, los grupos de población clave soportan una carga desproporcionada del VIH y reportan una utilización limitada de las pruebas del VIH, dada la generalización del estigma y la criminalización. En este contexto, el ADVIH puede servir como enfoque complementario para alcanzar a las poblaciones que reportan barreras al compromiso con los servicios de pruebas del VIH existentes y de rutina. En este estudio se distribuyeron 1839 ADVIH en Senegal, donde 1149 individuos participaron en una encuesta antes de recibir la prueba y 817 participaron en una encuesta después de recibir la prueba. En general, el 46.9% (536/1144) se realizó la prueba por primera vez y el 26.2% (300/1144) se habían realizado una prueba en el último año; el 94.3% (768/814) reportó utilizar el ADVIH y el 2.9% (19/651) reportó un resultado reactivo asociado con realizar la prueba por primera vez (*p* = 0.024). El ADVIH representa un enfoque que alcanzó a las personas que realizaron la prueba por primera vez y a quienes no se habían realizado la prueba recientemente. Los indicadores de implementación sugieren la importancia de aprovechar las estructuras comunitarias y los programas de distribución existentes.

## Introduction

Increasing coverage of HIV testing and early detection of seroconversion among people living with HIV is essential for effectively responding to the HIV pandemic. Early detection of HIV and initiation of antiretroviral therapy (ART) significantly reduces HIV-related morbidity and mortality, and can improve the quality of life for people living with HIV while also eliminating the risk of onward HIV transmission [1–3]. Similarly, awareness of one’s negative HIV serostatus is important for prioritizing prevention strategies especially in the context of increasing availability of pre-exposure prophylaxis (PrEP) [4, 5]. HIV self-testing (HIVST) is emerging as an important tool to potentially increase the uptake and the frequency of HIV testing in populations at increased risk for acquiring HIV such as key populations who may avoid HIV testing services because of stigma and criminalization of their sexual practices, orientation, or occupation, or even the criminalization of HIV transmission [6]. Approximately 48 countries have established an HIVST supportive policy and far more countries have policies under development, including several across sub-Saharan African [7, 8]. Given the rapid adoption of HIVST globally, WHO guidelines have been developed to support the implementation and scale-up of ethical, effective, acceptable, and evidence-based approaches to HIVST [9].

HIVST can potentially overcome barriers to HIV testing uptake and accessibility by placing the locus of control of testing on the individual, increasing confidentiality, and allowing members of marginalized and stigmatized groups to test in settings of privacy, safety, and with dignity [10]. Oral HIVST has been shown to improve HIV testing coverage and to be acceptable among diverse populations across varied settings [11–16]. However, there is currently limited evidence on acceptability of HIVST across Western and Central Africa despite the need to understand the acceptability and strategies for effective implementation across the region [8].

The West African country of Senegal is one of the countries in sub-Saharan Africa where an HIVST policy is currently under development [8]. Senegal has a concentrated HIV epidemic with a prevalence among adults of reproductive age consistently under 1%, and a high burden among specific key populations [17]. In Senegal, HIV disproportionately affects men who have sex with men (MSM), female sex workers (FSW) and people who inject drugs (PWID) with prevalence estimates of 23.5%, 3.3%, 10.2%, respectively [18, 19]. In Senegal, same-sex practices are criminalized and sex work for cisgender women is legal but highly regulated [20]. Stigma has been shown to be a barrier to uptake of HIV testing and accessing other HIV prevention and treatment services. In many places, there is stigma specifically associated with seeking HIV testing [21, 22]. Frequent or regular HIV testing may be perceived by healthcare providers as disclosing a stigmatized behavior, and stigma relating to access to health services among key populations has been reported to be high [18]. Low rates of testing may be affecting Senegal’s progress towards epidemic control among key populations and achieving the UNAIDS 90-90-90 targets for all [23]. While available data are limited, UNAIDS estimates that only 71% of adults living with HIV know their status, of which only 58% are receiving ART [24.] However, uptake of HIV services has been shown to be lower among key populations, with a recent study estimating that only 13% of MSM and 55% of FSW living with HIV reported to be aware of their seropositive status [18].

Given the HIV epidemic profile in Senegal and the limited uptake of HIV prevention and treatment services among key populations in the country, HIVST may represent an impactful strategy for increasing the uptake and coverage of HIV testing and accelerating progress towards achieving 90-90-90 goals. This study aimed to assess the acceptability of HIVST for key populations and people in their social and sexual networks and secondly, to assess the effectiveness of HIVST in reaching first-time testers. These results will inform appropriately scaled implementation of HIVST in Senegal and across West Africa.

## Methods

This is a pilot study which distributed HIVST kits through targeted venues and recruited individuals through convenience sampling to participate in pre and post HIVST socio-behavioral questionnaires.

### HIVST Distribution

OraQuick HIV Self-Test Kits (Orasure Technologies, Inc) were distributed to individuals in Dakar and Ziguinchor through venue and social network-based distribution. The HIVST kits included an OraQuick test device, written and pictorial step-by-step instructions, supplementary information on the test and HIV, and a referral card with information for confirmatory testing sites and study contacts. Instructions and supplementary information were provided in French and Wolof and adapted to the Senegalese context.

HIVST kit distribution and participant recruitment was led by study partner, Enda Santé, and aimed to reach populations with increased vulnerability of HIV acquisition and high levels of health care related stigma, including MSM, FSW, PWID, and clients of FSW. [20].

The venue-based approach for distribution and recruitment utilized directly assisted distribution of HIVST and was conducted through outreach to sex work venues, bars, nightclubs, hot spots, and mobile clinics, as well as health facilities that provide services to key populations. Venues were selected based on recommendations of community partners with previous experience in the communities, and leveraged existing programmatic activities. Directly assisted distribution of HIVST followed the WHO definition [9] and was led by trained distributors who provided pre-test instructions, test information, demonstration of proper HIVST use, and education on the importance for confirmatory testing, irrespective of a test reactivity. When possible, the participant was given the choice to either self-administer in a private space on-site with a peer educator available, or to take their HIVST kit away with them to test later.

A small sample of additional HIVST kits were distributed through social network-based unassisted distribution. The social network-based approach was focused on providing a primary recipient with one HIVST kit for themselves and two additional kits to distribute to individuals within their network. Social network-based distribution leveraged venue-based distribution to engage the primary HIVST recipient, who received the HIVST kits directly from the trained distributor. The primary recipient then distributed to secondary recipients through indirect, unassisted distribution as defined by WHO [9.] Secondary recipients only received written instructions and information contained within the HIVST kit.

### Data Collection

Convenience sampling was used to recruit individuals into the study at the time of HIVST kit distribution. Individuals receiving the HIVST kits through directly assisted venue-based distribution were asked if they wished to participate in a pre- and post-test survey. Data from social network-based distribution were only obtained from the primary recipient as follow up was not possible for the network-based HIVST kit recipients. Participants were eligible if they reported being 18 years of age or older; capable of and willing to provide informed consent; agreed to use the HIVST; and spoke Wolof and/or French. Participation was voluntary, and individuals could receive an HIVST kit regardless of survey participation. All pre- and post-test surveys were administered to eligible participants by trained interviewers. Among consenting participants, an interviewer administered pre-test surveys at the distribution site before HIVST utilization. Pre-test surveys captured information on demographic characteristics, HIV risk behaviors, HIV testing history, and motivation for testing.

Among individuals who opted to test at the HIVST distribution sites, the HIVST was collected through a test disposal box after self-administration and was read immediately. The result was logged to track the overall results observed, but not connected to the individual participant. This approach was used to compare aggregate level results to those self-reported in the post-tests. Post-test surveys assessing self-reported HIVST use and acceptability were conducted by phone two weeks after the HIVST kit distribution. Data were not obtained from secondary recipients.

Ethical review and approval were provided by the National Research Ethics Committee in Senegal and the Johns Hopkins School of Public Health Institutional Review Board.

### Measures

Key population characteristics were self-reported. Sex worker was defined as reporting exchanging sex for money or goods, and with more than half of income being from selling sex in the past 6 months. Male sex workers (MSW) were defined as sex workers above, as well as being assigned male sex at birth; and FSW were defined as sex workers as above and assigned female sex at birth. MSM was defined as being assigned the male sex at birth and ever having oral or anal sex with another man. Transgender women were defined using a two-step gender assessment of reporting male sex assigned at birth and gender identification as a woman. PWID were defined as ever having injected illicit drugs. Key population categories were not mutually exclusive. Key population was defined as meeting the criteria of at least one of the six key population categories.

First-time testers were defined as individuals who self-reported never having received an HIV test prior to the pre-test questionnaire. HIVST reactivity results were collected in two ways: 1. Results collected from used HIVST at the distribution sites; and 2. Self-reported HIVST results from those who participated in the post-test phone survey. Acceptability measures were informed by The Society for Implementation Research and Collaboration Indictor Review, however, have not yet been validated [25.]

### Statistical Analyses

Demographic characteristics and HIV testing history were determined from pre-test questionnaires. Logistic regression was used to assess the crude relationship between HIV testing history (first-time vs. previous testers), demographic characteristics, and HIV risk behaviors. Multiple multivariable logistic regression models were developed to separately assess each demographic characteristic, HIV testing history, HIV risk behaviors as primary predictors of first-time testers and adjusted for a priori demographic characteristics. Pearson’s Chi squared tests were used to assess the crude relationships between first-time testers and HIVST use and acceptability, as well as the relationships between self-reported HIVST result and use and demographic characteristics. A significance value of p < 0.05 was used for all analyses.

## Results

### Distribution and Study Participation

A total of 1839 HIVST kits were distributed between April 2017 to June 2018, and 62.5% (1149/1839) of recipients participated in the pre-test questionnaire before receiving the HIVST (Table 1). Among pre-test participants, 71.1% (817/1149) participated in the follow up post-test questionnaire.

**Table 1:** HIVST distribution and data collection summary in Senegal

	n/N	%
HIVST kits distributed	1839	100
Pre-test participants	1149/1839	62.5
Post-test participants among those who participated in the pre-test	817/1149	71.1
Received additional HIVST for secondary distribution among post-test respondents	48/810	5.9
Distributed HIVST for secondary distribution among post-test respondents	36/45	80.0
Gave HIVST to someone else, although did not receive additional HIVST for secondary distribution	9/730	1.2
HIVST results with positive reactivity among those collected at the distribution sites	76/1407	5.4

Among post-test respondents, 5.9% (48/810) had received additional HIVST kits for secondary, unassisted distribution, of which 80.0% (36/45) distributed the additional HIVST kits. Among individuals not provided additional HIVST kits for unassisted distribution, 1.2% (9/730) gave their HIVST kit to someone else.

### Demographic Characteristics

Among participants who completed the pre-test, 47.9% (539/1125) were in Dakar and 52.1% (586/1125) were in Ziguinchor (Table 2). Among pre-test participants, 25.3% (286/1130) were aged 18–24 years of age, 32.7% (370/1130) were 25–30 years, and 42.0 (474/1130) were 31 years and older. Overall, 52.9% (607/1148) reported female and 47.1% (541/1148) reported male sex at birth. Demographic characteristics of individuals who participated in the post-test questionnaire did not differ from the pre-test, except for region (p = 0.011).

**Table 2:** Demographic characteristics of individuals who participated in pre- and post- HIVST questionnaires

	Pre-test participants		Post-test participants		X^2^ p value to compare samples
	N = 1149		N = 817		
Demographic characteristics	n/N	%	n/N	%	
Region					0.011
Dakar	539/1125	47.9	437/813	53.7	
Ziguinchor	586/1125	52.1	376/813	46.3	
Age					0.947
18–24	286/1130	25.3	207/803	25.8	
25–30	370/1130	32.7	265/803	33.0	
31+	474/1130	42.0	331/803	41.2	
Sex at birth					0.226
Female	607/1148	52.9	454/816	55.6	
Male	541/1148	47.1	362/816	44.4	
Key populations^a^					
Key population (any)					0.451
Yes	370/1149	32.2	250/817	30.6	
No	779/1149	67.8	567/817	69.4	
Sex worker (all genders)					0.841
Yes	204/1085	18.8	148/772	19.2	
No	881/1085	81.2	624/772	80.8	
Female sex worker					0.772
Yes	155/1085	14.3	114/772	14.8	
No	994/1085	85.7	658/772	85.2	
Male sex worker					0.877
Yes	48/1085	4.4	33/772	4.3	
No	1101/1085	95.6	739/772	95.7	
Men who have sex with men					0.417
Yes	174/1149	15.1	113/817	13.8	
No	975/1149	84.9	704/817	86.2	
People who inject drugs					0.230
Yes	42/1131	3.7	22/807	2.7	
No	1089/1131	96.3	785/807	97.3	
Transgender women					0.800
Yes	20/1148	1.7	13/816	1.6	
No	1128/1148	98.3	803/816	98.4	
HIV testing history					
Recent testing for HIV					0.435
Never	536/1144	46.9	358/814	44.0	
Yes, but not in the last 12 months	308/1144	26.9	227/814	27.9	
Yes, within the last 12 months	300/1144	26.2	229/814	28.1	
First time testers					0.208
Yes	536/1144	46.9	358/814	44.0	
No	608/1144	53.1	456/814	56.0	

^a^Not mutually exclusive

### Key Populations

Among pre-test respondents, 32.2% (370/1149) self-reported membership of a key population group with 18.8% (204/1085) sex workers specifically, 14.3% (155/1085) FSW and 4.4% (48/1085) MSW; 15.1% (174/1149) MSM; 3.7% (42/1131) PWID; and 1.7% (20/1148) transgender women.

### First-Time Testers

Among pre-test respondents, 46.9% (536/1144) of participants were first-time testers, 26.9% (308/1144) had ever tested for HIV but not within the last 12 months, and 26.2% (300/1144) had tested within the last 12 months.

Among key populations, 36.8% (136/370) were first-time testers (Table 3). Among sex workers of all genders, 26.5% (54/204) were first time testers. Among FSW, 20.7% (32/155) were first-time testers, 27.7% (43/155) had tested but not in the last 12 months, and 51.6% (80/155) had tested in the last 12 months. Among MSW, 45.8% (22/48) were first-time testers. Overall, 46.0% (80/174) of MSM, 59.5% (25/42) of PWID, and 55.0% (11/20) of transgender women were first-time testers.

**Table 3:** HIV testing history among self-reported key populations in Senegal

	Total		HIV testing history						
Self-reported key population^a^			First-time tester		Yes, but not in the last 12 months		Yes, within the last 12 months		
	n/N	%	n/N	%	n/N	%	n/N	%	P value
Key population (any)									<0.001
Yes	370/1149	32.2	136/370	36.8	103/325	27.8	131/325	35.4	
No	779/1149	67.8	400/774	51.7	205/774	26.5	169/774	21.8	
Sex worker (all genders)									<0.001
Yes	204/1085	18.8	54/204	26.5	53/204	26.0	97/204	47.6	
No	881/1085	81.2	450/878	51.3	240/878	27.3	188/878	21.4	
Female sex worker									<0.001
Yes	155/1085	14.3	32/155	20.7	43/155	27.7	80/155	51.6	
No	930/1085	85.7	472/927	50.9	250/927	27.0	205/927	22.1	
Male sex worker									0.239
Yes	48/1085	4.4	22/48	45.8	9/48	18.8	17/48	35.4	
No	1037/1085	95.6	482/1034	46.6	284/1034	27.5	268/1034	25.9	
Men who have sex with men									0.923
Yes	174/1149	15.1	80/174	46.0	49/174	28.2	45/174	25.9	
No	975/1149	84.9	456/970	47.0	259/970	26.7	255/970	26.3	
People who inject drugs									0.184
Yes	42/1131	3.7	25/42	59.5	7/42	16.7	10/42	23.8	
No	1089/1131	96.3	500/1084	46.1	297/1084	27.4	287/1084	26.5	
Transgender women									0.242
Yes	20/1048	1.7	11/20	55.0	7/20	35.0	2/20	10.0	
No	1128/1148	98.3	525/1123	46.8	300/1123	26.7	298/1123	26.5	

^a^Not mutually exclusive

Among participants in Dakar, 42.2% (227/538) were first-time testers, and in Ziguinchor 52.6% (306/582) were first-time testers (Table 4). Among participants 18 to 24 years old, 62.8% (179/285) were first-time testers. Among participants ages 25–30, 46.0% (169/367) were first-time testers, and 38.0% (171/444) were first-time testers among those 31 years and older. Among participants assigned female sex at birth, 38.6% (233/604) were first-time testers compared to 56.2% (303/539) of participants assigned male sex at birth.

**Table 4:** Demographic characteristics, HIV testing history, motivation for HIV test use and associations with first-time testers in Senegal

Characteristics	Total		HIV testing history				X^2^ p value	OR	aOR*	95% CI	P value
			First-time tester		Individuals with testing history						
	n/N	%	n/N	%	n/N	%					
Region							0.001				
Dakar	539/1125	47.9	227/538	42.2	311/538	57.8		Ref	Ref		
Ziguinchor	586/1125	52.1	306/582	52.6	276/582	47.4		1.52	1.99	1.53,2.59	<0.001
Age							<0.001				
18–24	286/1130	25.3	179/285	62.8	106/285	37.2		2.75	2.84	2.07,3.90	<0.001
25–30	370/1130	32.7	169/367	46.0	198/367	54.0		1.39	1.32	1.00,1.76	0.063
31+	474/1130	42.0	171/444	38.0	293/473	62.0		Ref	Ref		
Sex at birth							<0.001				
Female	607/1148	52.9	233/604	38.6	371/604	61.4		Ref	Ref		
Male	541/1148	47.1	303/539	56.2	236/539	43.8		2.04	2.71	2.08,3.52	<0.001

HIV testing history	n/N	%	n/N	%	n/N	%	P value	OR	aOR**	95% CI	P value
Who suggested you get an HIV test?							0.056				
Sexual partner	66/1134	5.8	42/66	63.6	24/66	36.6		2.13	1.92	1.06, 3.49	0.032
Peer educator	359/1134	31.7	164/357	45.9	193/357	54.1		1.03	1.09	0.76, 1.54	0.648
Doctor	226/1134	19.9	101/224	45.1	123/206	54.9		1.00	1.01	0.68, 1.49	0.979
Family member	22/1134	1.9	14/22	63.6	8/22	36.4		2.13	2.61	1.01, 6.69	0.047
Friend	213/1134	18.7	97/213	45.5	116/213	54.5		1.01	0.95	0.63, 1.42	0.793
Other	248/1134	21.9	112/248	45.2	136/248	54.8		Ref	Ref		
In the last 12 months, worried about HIV							0.065				
Yes	837/1053	79.5	368/834	44.1	466/834	55.9		0.75	0.68	0.49, 0.94	0.021
No	216/1053	19.6	110/215	51.2	105/215	48.8		Ref			
Main reason for doing the HIVST today							<0.001				
Engaged in risky behavior	402/961	41.8	156/401	38.9	245/401	61.1		3.74	4.11	2.46.6.86	<0.001
Sex partner engaged in risky behavior	71/961	7.4	48/71	67.6	23/71	32.4		12.26	10.92	5.38, 22.17	<0.001
Had sex with someone knew/thought to be living with HIV	45/961	4.7	27/45	60.0	18/45	40.0		8.81	8.16	3.70, 17.98	<0.001
Condom broke or slipped	67/961	7.0	47/66	71.2	19/66	28.8		14.53	12.70	6.09, 26.51	<0.001
Someone suggested I get tested	199/961	20.7	116/197	58.9	81/197	41.1		8.41	9.32	5.33, 16.28	<0.001
Part of my regular testing pattern	165/961	17.2	24/165	14.6	141/165	85.5		Ref	Ref		
Other	12/840	1.3	5/12	41.7	7/12	58.3		4.20	1.28	0.24, 6.96	0.776

*adjusted for other demographic characteristics presented in this table

**adjusted for age, sex, and region

### Demographic Characteristics, HIV Testing History and HIV Risk Behaviors, and Associations with First-Time Testers

When adjusting for sex and age, region was associated with HIV testing history, with an increased odds of being a first-time tester in Ziguinchor (aOR: 1.99; 95%CI: 1.53, 2.59; *p* value: < 0.001) compared to Dakar (Table 4). Age was associated with HIV testing history with an increased odds of being a first-time tester among those 18 to 24 years old compared to 31 + (aOR: 2.84; 95CI %: 2.07, 3.90; p-value: < 0.001). Participants assigned male sex at birth had an increased odds of being a first-time tester compared to those assigned female sex (aOR: 2.71; 95CI %: 2.08, 3.52; p-value: < 0.001).

Among pre-test participants, 79.5% (837/1053) had been worried about their HIV status, which was negatively associated with being a first-time tester (aOR: 0.68; 95%CI: 0.49, 0.94; p-value: 0.021) (Table 4). A sexual partner (aOR: 1.92; 95%CI: 1.06, 3.49; p-value: 0.032) or a family member (aOR: 2.61; 95%CI: 1.01, 6.69; p-value: 0.047) suggesting getting tested for HIV were associated with reaching first-time testers compared to ‘other’ people suggesting. The reported primary reason for doing the HIV test was engagement in risky behavior (41.8%; 402/961), sexual partner engagement in risk behavior (7.4%; 71/961), had sex with someone who they thought or knew to be living with HIV (4.7%;45/961), condom failure (7.0%; 67/961), someone suggested to get tested (20.7%; 199/961), and part of a regular testing routine (17.2%; 165/961).

### Use of HIVST

Among post-test survey respondents, 94.3% (768/814) reported using the HIVST of which 43.5% (333/765) were first-time testers (Table 5). In total, 54.3% (363/668) used the HIVST at the distribution site and 45.7% (305/668) used the HIVST at home; and 88.9% (595/669) used the HIVST within 2 days. Among those who used the HIVST, 2.9% (19/651) reported a reactive result, and 2.0% (13/651) had an invalid result. Self-reported reactivity was associated with first-time testers (p = 0.024), and among those with a reactive result 63.2% (12/19) were first-time testers. Reported location of receiving the HIVST was associated with HIV testing history (p-value: < 0.001). Overall 10.3% (48/466) of those who reporting using the HIVST reported seeking follow up testing.

**Table 5:** Use and acceptability of HIVST and differences between first-time testers and individuals with HIV testing history in Senegal

	Total		First time testers		Individuals with testing history		
HIVST distribution and use	n/N	%	n/N	%	n/N	%	P value
Reported use of HIVST							0.390
Yes	768/814	94.3	333/765	43.5	432/765	56.5	
No	46/814	5.7	23/46	50.0	23/64	50.0	
Place of HIVST use							0.092
Home	305/668	45.7	147/302	48.7	155/302	51.3	
At distribution site	363/668	54.3	153/363	42.2	210/363	57.9	
Time of use after distribution							0.617
< 2 days	595/669	88.9	266/593	44.9	327/593	55.1	
> 2 days	74/669	11.1	35/73	48.0	38/73	52.1	
Where did you receive your HIV self-test?							<0.001
Hospital	260/742	35.0	130/259	50.2	129/259	49.8	
Community organization	78/742	10.5	24/78	30.8	54/78	69.2	
At a hotspot, bar, or community venue	194/742	26.2	95/192	49.5	97/192	50.5	
Mobile clinic	108/742	14.6	13/108	12.0	95/108	88.0	
Friend or family	102/742	13.8	69/102	67.7	33/102	32.4	
Self-reported result of HIVST							0.024
Negative	619/651	95.1	268/619	43.4	349/617	56.6	
Reactive	19/651	2.9	12/19	63.2	7/19	36.8	
Invalid	13/651	2.0	9/12	75.0	3/12	25.0	
Confirmed results of HIVST results							0.625
Yes	48/466	10.3	21/48	43.8	27/48	56.3	
No	418/466	89.7	197/415	47.5	218/415	52.5	
Acceptability of HIVST							
How comfortable did you feel using the HIVST?							<0.001
Comfortable	496/666	74.5	202/494	40.9	292/492	59.1	
Not comfortable	170/666	25.5	96/169	56.8	73/169	43.2	
How did you find the instructions?							0.427
Easy	576/669	86.1	263/575	45.7	312/575	54.3	
Not easy	93/669	13.9	38/92	41.3	54/92	58.7	
Would you recommend self-testing to others?							0.390
Yes	596/626	95.2	259/594	43.6	335/594	56.4	
No	30/626	4.8	15/29	51.7	14/29	48.3	
Do you think your friends and/or family would use an HIVST?							0.591
Yes	638/676	94.4	273/636	42.9	363/636	57.1	
No	38/676	5.6	18/38	47.4	20/38	52.6	
Since you receive the HIVST, did you discuss HIV testing with any sexual partners or friends?							0.582
Yes	244/797	30.6	104/244	42.6	140/244	57.4	
No	553/797	69.4	246/550	44.7	304/550	55.3	
Would you be comfortable asking your primary sexual partner to use an HIVST?							0.037
Yes	307/391	78.5	132/306	43.1	174/306	56.8	
No	84/391	21.5	47/84	56.0	37/84	44.1	
Would you be comfortable asking a casual sexual partner to use an HIVST?							0.218
Yes	150/228	65.8	72/149	48.3	77/149	51.7	
No	78/228	34.2	31/78	39.7	47/78	60.3	

### Acceptability of HIVST

Overall, 74.5% (496/666) participants reported being comfortable using the HIVST. In total, 86.1% (576/669) found the instructions easy to follow, and 94.4% (638/676) thought their family of friends would use the HIVST. After receiving the HIVST, 30.6% (244/797) discussed HIV testing with a sexual partner or friend. Among participants 78.5% (307/391) would be comfortable asking a primary sexual partner to use an HIVST, and 65.8% (150/228) would be comfortable asking a casual sexual partner to use an HIVST.

### HIVST Reactivity

Among post-test respondents reporting a reactive result, 42.1% (8/19) used the test on site, and 57.9% (11/19) used the HIVST at home (Table 6). Among those with a reactive HIVST, 57.9% (11/19) went for confirmatory testing and among those with an invalid test result none went for follow up testing. Among those with a reactive HIVST result, 84.2% (16/19) were male, 31.6% (6/19) were 18–24 years old, and 42.1% (8/19) were a self-reported member of a key population. Among HIVST kits collected at the distribution site, 5.4% (76/1407) had a positive reactivity (Table 1).

**Table 6:** HIVST result reactivity and association with use and demographic characteristics

	Reactive (N = 19)		Invalid (N = 13)		Not reactive (N = 619)		X^2^ P value
	n/N	%	n/N	%	n/N	%	
Place of HIVST use							0.002
Home	11/19	57.9	12/13	92.3	274/616	44.5	
At distribution site	8/19	42.1	1/13	7.7	3412/616	55.5	
Confirmed results of HIVST results							<0.001
Yes	11/19	57.9	0/11	0.0	37/423	8.8	
No	8/19	42.1	11/11	100.0	386/423	91.4	
Sex							<0.001
Female	3/19	15.8	3/13	23.1	350/619	56.5	
Male	16/19	84.2	10/13	76.9	269/619	43.5	
Age							0.327
18–24	6/19	31.6	1/13	7.7	56/610	25.6	
25–30	9/19	47.4	6/13	46.2	210/610	34.4	
31+	4/19	21.1	6/13	46.2	244/610	40.0	
Key population							0.326
Yes	8/19	42.1	3/13	23.1	167/619	73.0	
No	11/19	57.9	10/13	76.9	452/619	27.0	

## Discussion

This study demonstrates that HIVST can effectively engage first-time testers at risk for HIV in Senegal, including key populations, cisgender men, and young adults. Expanding access to HIVST may increase the coverage and frequency of HIV testing and thus have an important role in linking people living with HIV to diagnosis and treatment services and potentially mitigating the HIV epidemic in Senegal. Overall history of HIV testing as well as frequency of testing remains low among key populations, as well as among young adults in their social and sexual networks in Senegal. HIVST result reactivity was associated with first-time testing, and among those who tested with an HIVST, acceptability was high for both first-time testers and those reporting previous HIV testing. However, consistent with some earlier studies, confirmatory testing and linkage to care was a challenge during the implementation of HIVST in Senegal [26, 27].

This study highlights that HIVST was able to reach a large proportion of individuals, and in particular key populations, who had never received an HIV test as well as those who had not tested recently. Notably, approximately half of MSW, MSM, PWID, and transgender women reached through HIVST reported not having tested for HIV. Few programs currently exist to provide tailored health services to PWID and transgender women in Senegal, and this study suggests that HIVST may provide an opportunity for PWID and transgender women to increase uptake of testing in this context [28]. The proportion of first-time testers among FSW was lower, suggesting comparatively higher coverage of HIV testing among FSW than other key populations [18]. Sex work is legal in Senegal but is strictly regulated through a registration process for sex workers which includes requirements for HIV testing [20]. Despite this, frequency of testing among FSW is low compared to the recommended guidelines for HIV testing among key populations. Many FSW are not legally registered for sex work in Senegal, and these data suggest potential barriers to traditional testing approaches within challenging environments [18].

This small scale implementation of HIVST leveraged existing programs and networks working with key populations to distribute HIVST. Despite available services and programs in Senegal, HIVST was able to reach a large proportion of first-time testers in this study. Therefore, HIVST represents a promising new approach to increase coverage and uptake of HIV testing through leveraging current programs. However, adoption and integration of HIVST into existing programs will require a revision of the current HIV testing targets for programs in Senegal. HIVST indicators have been incorporated into the PEPFAR Monitoring, Evaluation, and Reporting (MER 2.0) Indicator Reference Guide representing appropriate indicators for collection in HIV testing programs [29]. Notably, the HIV testing yield for programs may decrease if HIVST are included though there will be a lower cost per test offered [30].

First-time testers were associated with HIVST result reactivity in this study, with the majority of self-reported reactive results being among first-time testers. These findings suggest the potential effectiveness of HIVST in increasing HIV diagnosis among those living with HIV in Senegal and not accessing traditional testing services. Additionally, acceptability was overall high among individuals who participated in the post-test survey, as shown in other settings [16, 31, 32]. However, one quarter of participants reported that they were not comfortable using the HIVST, which highlights the need to better understand how to improve comfort during testing. Use and acceptability of HIVST was overall not significantly different between first-time testers and those with a testing history for most measures in this study. These results suggest potential for sustained uptake among both new and returning users. Contrarily, other studies have found that acceptability was influenced by prior HIV testing [33].

Although acceptability of HIVST has been high in other studies, consistent evidence on confirmatory testing and linkage to care similarly remain sub-optimal [26, 27]. In this study, confirmatory testing was low, with approximately two-thirds of those with reactive results, and none with invalid results reporting confirmatory testing. A recent study in Zambia found that individuals who had not previously tested for HIV were negatively associated with intention to linkage to care after HIVST [33]. Therefore, there is a need to better understand implementation strategies for linkage to care, especially for first-time testers. Preferred methods for follow up have varied across studies [33, 34]. Community-based confirmation testing was preferred to facility-based testing in Zambia and Malawi [35]. Some studies have shown success in linkage to care through active follow up, however another study found active support for linkage was less important to individuals than other attributes of confirmation testing locations [35]. HIVST strategies in Senegal may require more active mechanisms for follow up and support to improve linkage to confirmatory testing and care. Notably, young adults in this study had a higher odds of being first-time testers, suggesting traditional testing services are not currently reaching this group in Senegal. HIV incidence among adolescents and young adults is high globally, however uptake of HIV services is low [36]. In particular, HIV incidence is generally highest among young MSM in countries with age-disaggregated incidence data [37–39]. The emergence of social media and technology to engage young adults and though social and sexual networks may provide an avenue for increasing uptake of HIV testing services for these populations [40]. Mobile phone apps have also been shown to be acceptable among young MSM in other settings and have been used to assess risk and coordinate HIVST distribution [41–43]. HIVST web-based delivery has been acceptable across settings, including sub-Saharan Africa, and may provide further opportunity to increase uptake and frequency of testing among young MSM [41–43]. Mobile technology may also be an opportunity to reach individuals in rural areas where program coverage and access to services is less, such as the region of Ziguinchor [44].

Several limitations should be considered in this study. Participation in the pre- and post-test questionnaires was voluntary and may not represent the full sample of individuals who participated in HIVST distribution. The results may therefore be subject to bias. Participants who received HIVST through network distribution were not captured in data collection and are not represented in this analysis. Disclosure of key population status as well as positive reactivity from the HIVST were low in self-reported measures of this study. The distribution strategy prioritized members of key populations and worked closely with existing programs providing services to these populations. However, only one-third of the study sample self-reported key population status. Therefore, it may be that HIVST reached individuals who may not currently be at high risk of HIV, in which case there is a need to consider strategies to more effectively target key populations. Alternatively, key population status may have been underreported, in which case HIVST was able to reach individuals unwilling to disclose their key population-related behavior and less integrated into the key population networks [45]. Additionally, there was a discrepancy between the proportion of reactive HIVST collected at the distribution sites and those who self-reported reactive results during posttest questionnaire. Although these figures cannot be linked or compared directly, it may suggest either underreporting of reactive test results, or possibly greater loss to follow up for posttest questionnaire among individuals with a reactive HIVST.

## Conclusions

In Senegal, key populations bear a disproportionate burden of HIV, and report limited uptake of existing HIV testing services given pervasive stigma and criminalization. In these contexts, HIVST may represent a complementary approach to reach populations reporting barriers to engagement with existing and routine HIV testing services. These data suggest the potential impact that HIVST could have in complementing existing HIV testing services by reaching a diverse group of first-time HIV-testers as well as those who have not tested recently in Senegal. This small-scale implementation further suggested the importance of leveraging existing structures and programs for distribution. Moreover, since HIVST has the potential to disrupt traditional testing approaches, sustained engagement with government and community stakeholders is needed to inform optimal implementation strategies of HIVST.
